# Implementing Early Phase Treatments for COVID-19 in Outpatient Settings: Challenges at a Tertiary Care Center in Italy and Future Outlooks

**DOI:** 10.3390/idr14030037

**Published:** 2022-04-25

**Authors:** Tommaso Manciulli, Filippo Lagi, Anna Barbiero, Marco Fognani, Nicoletta Di Lauria, Costanza Malcontenti, Costanza Fiorelli, Michele Spinicci, Vega Ceccherini, Paola D’Onofrio, Manuela Angileri, Francesca Malentacchi, Michele Cecchi, Gian Maria Rossolini, Matteo Tomaiuolo, Lorenzo Zammarchi, Alessandro Bartoloni

**Affiliations:** 1 Department of Experimental and Clinical Medicine, University of Florence, 50134 Florence, Italy; tommaso.manciulli@unifi.it (T.M.); filippo.lagi@unifi.it (F.L.); anna.barbiero@unifi.it (A.B.); marco.fognani@unifi.it (M.F.); michele.spinicci@unifi.it (M.S.); gianmaria.rossolini@unifi.it (G.M.R.); lorenzo.zammarchi@unifi.it (L.Z.); 2 Unit of Infectious and Tropical Diseases, Careggi University Hospital, 50134 Florence, Italy; dilaurian@aou-careggi.toscana.it (N.D.L.); malcontentic@aou-careggi.toscana.it (C.M.); fiorellic@aou-careggi.toscana.it (C.F.); 3 Hospital Management Unit, Careggi University Hospital, 50134 Florence, Italy; ceccheriniv@aou-careggi.toscana.it (V.C.); donofriop@aou-careggi.toscana.it (P.D.); tomaiuolom@aou-careggi.toscana.it (M.T.); 4 Hospital Pharmacy, Careggi University Hospital, 50134 Florence, Italy; angilerim@aou-careggi.toscana.it (M.A.); cecchim@aou-careggi.toscana.it (M.C.); 5 Clinical Microbiology and Virology Unit, Careggi University Hospital, 50134 Florence, Italy; malentacchif@aou-careggi.toscna.it

**Keywords:** SARS-CoV-2, monoclonal antibodies, molnupiravir, nirmatrelvir/ritonavir, sotrovimab, remdesivir

## Abstract

We present a brief commentary illustrating the current COVID-19 outpatient treatment options in Italy. We also report our experience setting up a service dedicated to these patients in the wake of the rise in COVID-19 cases observed in January 2022. We also gathered data on the daily costs faced by our outpatient service, based at a tertiary care center located in Florence, Italy. We present them with some considerations on future outlooks on the use of outpatient treatment in COVID-19.

## 1. Introduction

Since the beginning of the COVID-19 pandemic, early treatment options have been needed. At first, hydroxychloroquine and lopinavir/ritonavir were employed [1], and some countries, including Italy, used them for treatment in hospitalized and at-home patients. Both options showed no beneficial effects and were dropped from national and international recommendations for COVID-19 treatment [2,3,4]. Monoclonal antibodies (mAbs) first demonstrated a reduction in hospitalizations or deaths by 70–87% among outpatients [1], as well as in the prevention of infection after exposure to SARS-CoV-2 [2]. Casirivimab/imdevimab and tixagevimab/cilgavimab have also been approved for pre-exposure prophylaxis [2]. However, intravenous administration has limited mAbs in many settings, especially in resource-limited countries. Some mAbs have also been tested for subcutaneous and/or intramuscular use, with initial success [5,6]. Sadly the advent of the Omicron variant, dominant in Europe since the end of January [7], has caused casirivimab/imdevimab and bamlanivimab/etesevimab to become inefficacious [2], as shown by preliminary in vitro data [2,8]. This led to a drop in the use of both mAbs [2]. Currently sotrovimab is the only mAb available in Italy which retains efficacy against the Omicron BA.1 sublineage [2]. This was thought to be possible due to the targeting of a highly conserved epitope, functionally retained as SARS-CoV-2 evolves [9]. However, according to recent data showing reduced in vitro neutralization activity against the Omicron BA.2 subvariant, the authorized dose of sotrovimab is unlikely to be effective against this variant. Due to these data, sotrovimab is no longer authorized by Food and Drug Administration to treat COVID-19 in the United States where the BA.2 subvariant is the predominant circulating variant [2,8,10]. Sotrovimab is currently available in Italy based on an emergency use authorization in Europe. However, the rapid diffusion of BA.2 subvariant also in Europe currently limits the use of this mAb. In the matter of fact in Italy the subvariant BA.2 increased from 1% of sequenced cases on 17th January 2022 to 87% on 4th April 2022 (last data available at the time of writing) [11]. First results from a trial for intramuscular use have shown interesting results, opening the door to the at-home administration of this mAb [6]. Recent, pre-published data suggests that imdevimab retains part of its activity on the BA.2 subvariant, although other reports have shown conflicting evidence. Bebtelovimab, a mAb not approved in Europe, seems to retain efficacy on all Omicron subvariants. Like the other mAbs mentioned here, trials for this compound were conducted on unvaccinated patients and at a time when the wild type or delta variants were dominant [12]. Further data will be needed to confirm these preliminary reports.

Antivirals have also recently found a place as early treatment option. Remdesivir (RMD), an intravenous direct-acting adenine nucleotide prodrug RNA polymerase RNA-dependent inhibitor, was conceived as a treatment for Ebola and shown to be active against SARS-CoV-2. An early five-day course of RMD reduces morbidity and mortality in inpatients [1,2]. Treatment after seven days from symptoms’ onset showed no difference in morbidity and mortality outcomes [1]. Using a three-day course of RMD in outpatients reduced the risk of hospitalization or death by 87% [2,13]. However, the need for multiple doses and kidney and liver function evaluation leads to logistical constraints [13]. Therefore, an ideal treatment option for early COVID-19 should have these characteristics (1) orally administered, (2) dispensable by general practitioners (GPs) (3) low pill burden with minor interactions. Two oral treatments are available: nirmatrelvir/ritonavir (a protease inhibitor) and molnupiravir (a prodrug of the synthetic nucleoside derivative N4-hydroxycytidine) [2,14]. The latter has success rate inferior to mAbs (30% hospitalization reduction) [2,14] and requires eight pills/day. The former has a lower pill burden and had results comparable to mAbs (88% reduction in hospitalizations/death), but is burdened by relevant drug interaction issues, mainly related to ritonavir [2,15]. Here, we present an account of the challenges faced in the first month of increased outpatient treatment at a tertiary care center located in Florence, Italy.

## 2. Materials and Methods

### 2.1. Setting and Treatment Allocation

**Figure 1:**
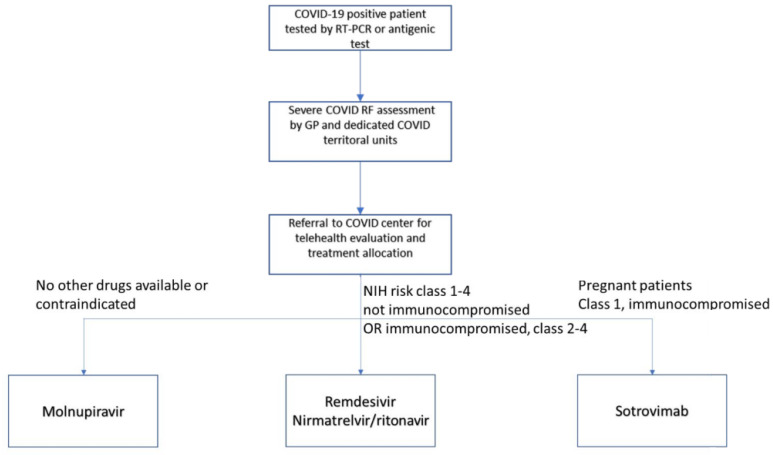
Flowchart for treatment allocation at our COVID-19 outpatient service. Risk classes were established according to the National Institutes of Health (NIH) statement on treatment allocations [2].

Two rooms have been dedicated to outpatients at our center since March 2021. Requests rose at the start of January 2022 as referrals from general practitioners and territorial physicians increased. The outpatient service welcomes up to twelve patients per day for intravenous treatments at full capacity. Oral treatments are dispensed as needed. Starting the service required efforts from both the Infectious Diseases (ID) and Emergency Department (ED), as nurses from both units were employed. We also rely on efforts from a centralized office dedicated to patient transportation in case they cannot come autonomously. We have also transiently integrated the rapid determination of variants during the rise of Omicron cases to allocate treatments better. One ID specialist, one resident, and one nurse staffed the clinic. During a sotrovimab shortage, we administered treatments based on patient risk factors (Figure 1). Sotrovimab was reserved for higher-risk patients or those for whom multiple-day treatments are unfeasible. Comorbidities such as severe hepatic and renal disfunction as well as interactions with other drugs are also considered [2].

### 2.2. Cost Estimation

Costs were estimated based on previous reports present in scientific articles and media outlets [16,17,18]. Costs for laboratory analyses, staff, and transport services were estimated based on hospital prices provided by the Careggi University Hospital management unit. The number of daily administered treatments was estimated by using daily administered treatments in the first ten days of activity of the outpatient service. Overheads are calculated as 26% of all in-hospital expenses excluding drugs.

## 3. Results

Current direct costs for the treatment of one patient depending on the use of mAbs, RMD, nirmatrelvir/ritonavir, or molnupiravir are reported in Table 1. While current reports place the cost of a cycle of molnupiravir at around EUR 700 for a complete cycle, monoclonal antibodies have been estimated to cost around EUR 1250 per dose. At our center, COVID-19 hospitalizations in medical ward have cost between EUR 1000 and EUR 60.700, with a mean expense of EUR 6947.68.

## 4. Discussion

Overall, all outpatient options come with challenges, and these may depend on the setting chosen for administration. Nirmatrelvir/ritonavir requires dose adjustment based on the kidney function. The use of ritonavir also causes numerous interactions with other drugs and these are well known by ID physicians since the drug was used to treat HIV patients [2], while the presence of kidney failure with a glomerular filtrate of less than 60 mL/min is an absolute contraindication to the use of molnupiravir [2]. In most regions of Italy, they are both available only by prescription of physicians expert in COVID-19 management headquartered in a hospital setting. None of the options currently available cover the treatment of young children. Sotrovimab and RMD are on-label for ages older than 12 years with a weight of at least 40 kg. Oral antivirals are approved for patients older than 18. Pregnant and breastfeeding patients can be treated with sotrovimab, RMD, and nirmatrelvir/ritonavir according to NIH guidelines [2], although nirmatrelvir/ritonavir is not recommended in Italy for this population. Molnupiravir is contraindicated in pregnant women due to potential mutagenicity, and men have to abstain from unprotected sex for the three months following this treatment [2]. Physicians must monitor patients treated with mAbs for one hour after administration in Italy. This introduces the need for dedicated spaces for the administration of mAbs and makes home administration difficult, although this has been done in other countries [19]. Some centers chose a direct administration of treatments in ED. However, EDs are often understaffed, and the technical time of two hours for administration makes this an exceptional circumstance in most centers in Italy. Multiple accesses for RMD administration are even more challenging. Therefore, in our country, the use of outpatient treatment options requires intense efforts from hospital-based personnel, including a constant dialogue with territorial health services. It has often meant reallocating physicians from required COVID-19 wards to outpatient services.

Our center can count on resources smaller hospitals might not have, and hospitalization surges can complicate their allocation. Future efforts should include setting up a protocol for early treatments in at-home settings. The cooperation with territorial teams managing COVID-19 patients could be crucial for a timelier treatment of patients.

## Figures and Tables

**Table 1:** The daily costs faced by our COVID-19 outpatient service. Costs of drugs employed for treatment vary depending on the number of treatments allocated to patients.

Item	Unit cost	Per Day/Unit	Daily Cost (EUR)
**Ambulance transport (EUR/km)**	21.48	35 km	751.8
Overheads *	-	-	228.02
**Senior physician salary (EUR/h)**	60	1 staff physician	600
**Resident salary (EUR/h)**	8.3	1 resident	83
**Nursing staff salary (EUR/h)**	30	1 nurse	60
**Patient nursing (EUR/patient)**	10	12 patients	120
**Fixed costs (Total)**	-	-	1842
**Remdesivir (EUR/dose)**	337	4–7 doses	1348–2359
**Sotrovimab (EUR/treatment)**	1250	3–4 treatments	3750–5000
**Molnupiravir (EUR/treatment)**	700	0–3 treatments	0–2100
**Nirmatrelvir/ritonavir (EUR/treatment)**	700	0–3 treatments	0–2100
**Laboratory costs (EUR/patient)**	14	4–7 treatments	14
**Total costs (total)**	2393.78	-	8976.82–15,257.82

* Overheads are calculated as 26% of all in-hospital expenses excluding drugs.

## Data Availability

Not applicable.
